# Association of Cerebrospinal Fluid Tumor DNA Genotyping With Survival Among Patients With Lung Adenocarcinoma and Central Nervous System Metastases

**DOI:** 10.1001/jamanetworkopen.2020.9077

**Published:** 2020-08-04

**Authors:** Yang-Si Li, Mei-Mei Zheng, Ben-Yuan Jiang, Hai-Yan Tu, Jin-Ji Yang, Xu-Chao Zhang, Yi-Long Wu

**Affiliations:** 1 Guangdong Lung Cancer Institute, Guangdong Provincial Key Laboratory of Translational Medicine in Lung Cancer, Guangdong Provincial People’s Hospital and Guangdong Academy of Medical Sciences, School of Medicine, South China University of Technology, Guangzhou, China

## Abstract

**Importance:**

Owing to the improvement of systemic therapies for lung cancer, patients live longer, but the incidence of central nervous system (CNS) metastases also increases. Cerebrospinal fluid (CSF) has been proven better than plasma to reveal unique genetic profiling of intracranial metastases. How genetic alterations in CSF are associated with the prognosis of this heterogeneous patient group remains elusive.

**Objective:**

To examine the association of molecular alterations in CSF with the survival of patients with a diagnosis of lung adenocarcinoma and CNS metastases.

**Design, Setting, and Participants:**

This retrospective cohort analysis of 94 patients with advanced lung adenocarcinoma and CNS metastases was conducted from July 1, 2016, to July 31, 2018. Patients’ CSF samples were collected, and next-generation sequencing of CSF circulating tumor DNA was performed.

**Main Outcome and Measures:**

The main outcome was survival after diagnosis with CNS metastases. Genotyping of CSF circulating tumor DNA was studied to examine its association with the clinical outcomes of patients with CNS metastases.

**Results:**

Of the 94 patients (49 male; mean [SD] age, 53 [1] years) with lung adenocarcinoma and CNS metastases evaluated, 79 harbored an *EGFR* variant. The most common genes seen in CSF were *EGFR* (79 [84.0%]), *TP53* (57 [60.6%]), *MET* (23 [24.5%]), *CDKN2A* (22 [23.4%]), *MYC* (20 [21.3%]), *NTRK1* (19 [20.2%]), and *CDK6* (15 [16.0%]). Cluster analysis identified 5 molecular subtypes of CNS metastases. Patients in cluster I had the shortest median survival after diagnosis of CNS metastases compared with each of the other clusters (cluster I, 7.5 months; cluster II, 55.7 months; cluster III, 17.9 months; cluster IV, 27.9 months; cluster V, 21.0 months) and significantly increased risk of death compared with patients in the other clusters (cluster II: hazard ratio [HR], 4.95; 95% CI, 1.50-16.41; *P* = .009; cluster III: HR, 4.75; 95% CI, 1.49-15.12; *P* = .008; cluster IV: HR, 6.38; 95% CI, 1.76-23.09; *P* = .005; cluster V: HR, 5.42; 95% CI, 1.63-17.98; *P* = .006). The genetic profiles of cluster I were characterized by a high detection rate of *CDK4* (9 of 9 [100%]), *TP53* (8 of 9 [88.9%]), *MET* (7 of 9 [77.8%]), and *CDKN2A* (7 of 9 [77.8%]). For those with *EGFR* variants, coalterations with *CDK4* (HR, 2.02; 95% CI, 1.03-3.96; *P* = .04), *CDK6* (HR, 2.52; 95% CI, 1.32-4.83; *P* = .005), and *MYC* (HR, 2.24; 95% CI, 1.21-4.15; *P* = .01) were associated with poor outcomes.

**Conclusions and Relevance:**

Patients with a diagnosis of lung adenocarcinoma and CNS metastases experienced heterogeneous survival outcomes based on genetic profiling in CSF. These data suggest that CSF might facilitate risk stratifying CNS metastases into appropriate outcomes and provide reference for further clinical study.

## Key Points

**Question:**

What is the association of molecular alterations in cerebrospinal fluid with clinical outcomes of patients with a diagnosis of lung adenocarcinoma and central nervous system metastases?

**Findings:**

In this cohort study of 94 patients, next-generation sequencing of cerebrospinal fluid showed that 5 molecular subtypes were associated with survival; the molecular subtype with abundant genetic alterations was associated with the shortest survival and increased risk of death. Specifically, *EGFR* variants coaltered with *CDK4*, *CDK6*, *MYC*, and *MET* were associated with poor outcomes.

**Meaning:**

Based on genetic profiling in cerebrospinal fluid, this population experienced heterogenous survival outcomes, and some specific molecular alterations were associated with prognosis; therefore, as liquid biopsy is performed of central nervous system metastases, cerebrospinal fluid may facilitate risk stratifying central nervous system metastases into appropriate outcomes.

## Teaser

This cohort study examines the association of molecular alterations in cerebrospinal fluid (CSF) with the survival of patients with a diagnosis of lung adenocarcinoma and central nervous system (CNS) metastases.

## Introduction

Owing to the improvement of systemic therapies for lung cancer, patients live longer, but the incidence of central nervous system (CNS) metastases also increases. Cerebrospinal fluid (CSF) has been proven better than plasma to reveal unique genetic profiling of intracranial metastases. Recent research has indicated that CSF could be used as a liquid biopsy of CNS metastases because CSF reveals unique genetic profiles of patients with non–small cell lung cancer and CNS metastases.^1,2,3^ However, the clinical effect of the abundant genetic alterations in CSF remains elusive. Thus, the present study aims to explore the molecular alterations revealed in CSF samples and their associations with survival of patients with lung adenocarcinoma and CNS metastases.

## Methods

This retrospective cohort study was conducted at Guangdong Lung Cancer Institute, Guangzhou, China, from July 1, 2016, to July 31, 2018, among 94 patients with late-stage lung adenocarcinoma and a diagnosis of CNS metastases (leptomeningeal metastases [LM] and brain metastases [BM]). Leptomeningeal metastases were diagnosed based on tumor cells detected in CSF samples or detected by leptomeningeal enhancement in brain magnetic resonance imaging. Brain metastases were diagnosed based on metastatic lesions detected by brain magnetic resonance imaging. All patients provided written informed consent, and the study protocol was approved by the Research Ethics Committee of Guangdong Provincial People’s Hospital. This study followed the Strengthening the Reporting of Observational Studies in Epidemiology (STROBE) reporting guideline.

All patients underwent lumbar puncture, and circulating tumor DNA was extracted from CSF samples and profiled by a 168-gene targeted next-generation sequencing panel; the preparation of CSF circulating tumor DNA and a next-generation sequencing library and sequencing data analysis were fully described in a previous study.^3^ Survival was calculated from the day of diagnosis of CNS metastases until death or the last follow-up date. Survival status was classified as censored if a patient was unavailable for follow-up or survived beyond the last follow-up. Follow-up was administratively censored on December 15, 2018.

### Statistical Analysis

Patients were divided into several groups using unsupervised hierarchical cluster analysis using R, version 3.3.1 (R Foundation for Statistical Computing).^4^ The variant status of each gene was valued as 0 or 1, and genes with a frequency greater than 10% were included. Kaplan-Meier survival curves were used to evaluate survival. The association between survival and coaltered genes (detected frequency >5%) with *EGFR* (GenBank NG_007726) variants was determined by logistic regression analysis. The coaltered genes and the clinical characteristics of the patients were included in the Cox proportional hazards regression models, and hazard ratios (HRs) and 95% CIs were calculated to determine the survival difference. All *P* values were 2-sided and were considered statistically significant at *P* < .05. SPSS for Windows, version 22.0 (SPSS Inc) was used for all statistical analysis; Prism, version 8.0 (GraphPad Software) was used for making graphics.

## Results

Of the 94 patients (49 male; mean [SD] age, 53 [1] years) with lung adenocarcinoma and a diagnosis of CNS metastases, 70 received a diagnosis of LM, and the remaining 24 received a diagnosis of BM. All patients had at least 1 molecular alteration detected in a CSF sample. The demographic and clinical characteristics of the included patients are shown in Table 1. The most common genes seen in CSF were *EGFR* (79 [84.0%]), *TP53* (GenBank NG_017013) (57 [60.6%]), *MET* (GenBank NG_008996) (23 [24.5%]), *CDKN2A* (GenBank NG_007485) (22 [23.4%]), *MYC* (GenBank NG_007161) (20 [21.3%]), *NTRK1* (GenBank NG_007493) (19 [20.2%]), and *CDK6* (GenBank NG_015888) (15 [16.0%]) (eFigure in the Supplement).

**Table 1.** Demographic and Clinical Characteristics of Included Patients

Characteristic	Cluster I (n = 9)	Cluster II (n = 19)	Cluster III (n = 29)	Cluster IV (n = 11)	Cluster V (n = 26)	Total (N = 94)
Sex						
Male	4	15	13	8	9	49
Female	5	4	16	3	17	45
Age, y						
<55	5	6	20	3	16	50
≥55	4	13	9	8	10	44
Extracranial metastases						
Yes	8	15	20	11	22	76
No	1	4	9	0	4	18
ECOG PS						
0 or 1	7	17	23	8	19	74
>1	2	2	6	3	7	20
Histologic characteristics, adenocarcinoma	9	19	29	11	26	94
CSF cytologic findings						
Positive	6	14	16	7	15	58
Negative	3	5	12	4	9	33
Unavailable	0	0	1	0	2	3
Brain MRI results						
Positive	5	6	15	6	12	44
Negative	2	11	9	4	12	38
Unavailable	2	2	5	1	2	12
Progression status at CSF collection						
Treatment naive	1	5	3	0	5	14
PD to first- or second-generation TKI	6	10	11	9	15	51
PD to osimertinib	2	1	13	0	4	20
Others^a^	0	3	2	2	2	9

Abbreviations: ECOG PS, Eastern Cooperative Oncology Group performance score; CSF, cerebrospinal fluid; MRI, magnetic resonance imaging; PD, progression of disease; TKI, tyrosine kinase inhibitor (gefitinib, erlotinib, or afatinib).

^a^ PD to avitinib or AZD3759 and no targetable driver variants detected.

### Molecular Subtypes of Patients With Lung Adenocarcinoma With LM or BM Distinguished by CSF

**Figure 1.**
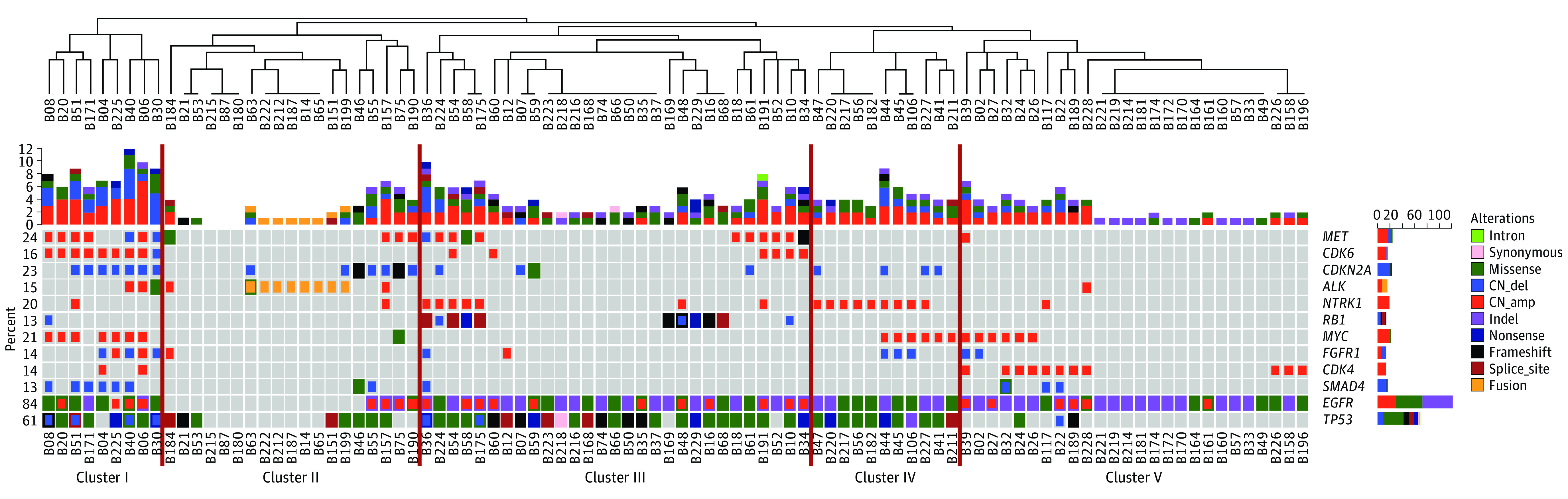
Heatmap of Unsupervised Cluster Analysis of Cerebrospinal Fluid Tumor DNA and Patient Survival The vertical red lines indicate the 5 clusters.

The median survival of the entire cohort was 19.3 months (95% CI, 15.4-23.2 months). Unsupervised hierarchical cluster analysis was used to identify 5 major molecular subtypes associated with CSF samples obtained from patients with CNS metastases (subtype cluster 1, 9 cases; cluster II, 19 cases; cluster III, 29 cases; cluster IV, 11 cases; cluster V, 26 cases) (Figure 1).

**Figure 2.**
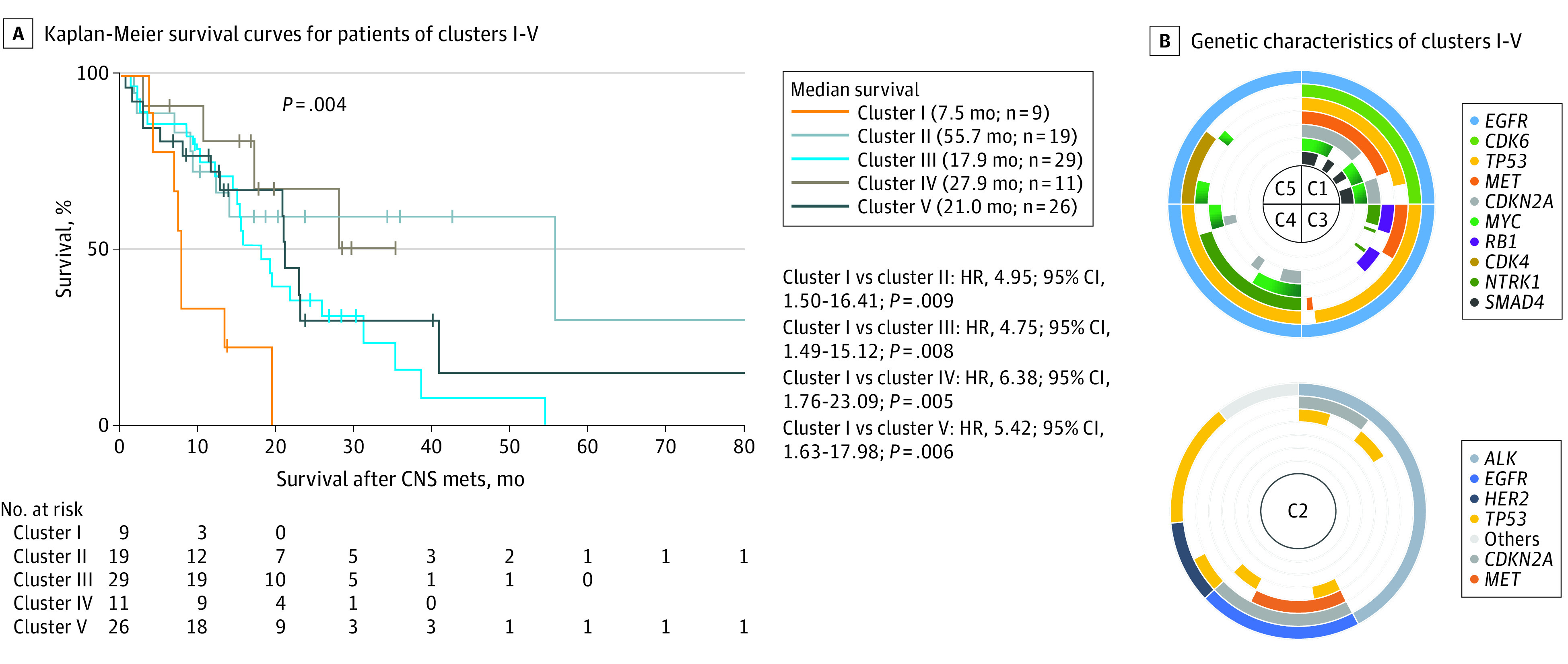
Characteristics of Patients in Clusters I to V A, Kaplan-Meier survival curves. B, Genetic characteristics. C indicates cluster, CNS mets, central nervous system metastases; and HR, hazard ratio.

The demographic and clinical characteristics of these molecular subtypes are shown in Table 1. Patients in cluster I had a significantly shorter median survival than did the patients in each of the other clusters (cluster I, 7.5 months; cluster II, 55.7 months; cluster III, 17.9 months; cluster IV, 27.9 months; cluster V, 21.0 months). With the shortest median survival, the patients in cluster I had a significantly increased risk of death compared with those in cluster II (HR, 4.95; 95% CI, 1.50-16.41; *P* = .009), cluster III (HR, 4.75; 95% CI, 1.49-15.12; *P* = .008), cluster IV (HR, 6.38; 95% CI, 1.76-23.09; *P* = .005), or cluster V (HR, 5.42; 95% CI, 1.63-17.98; *P* = .006) (Figure 2A).

### Specific Genetic Signatures of 5 Molecular Subtypes

**Figure 3.**
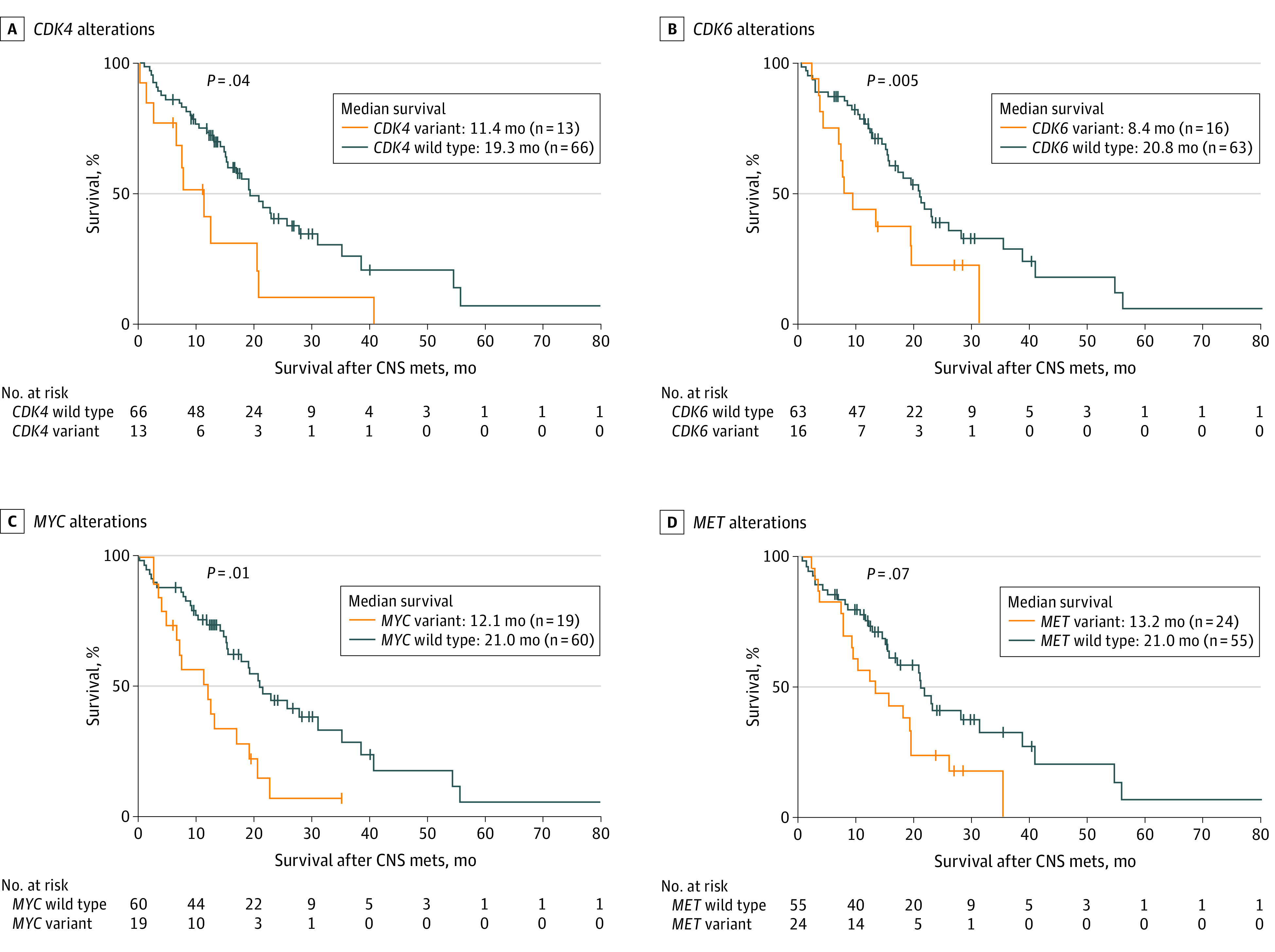
Coaltered Genes in CSF Linked to Survival of Patients With an *EGFR* Variant and Lung Adenocarcinoma With CNS Metastases A, *CDK4* alterations (HR, 2.02; 95% CI, 1.03-3.96). B, *CDK6* alterations (HR, 2.52; 95% CI, 1.32-4.83). C, *MYC* alterations (HR, 2.24; 95% CI, 1.21-4.15). D, *MET* alterations (HR, 1.71; 95% CI, 0.96-3.05). CNS mets indicates central nervous system metastases; CSF, cerebrospinal fluid.

Patients in clusters I, III, IV, and V all harbored *EGFR* variants, while most patients in cluster II carried *ALK* (GenBank NG_009445) fusion (Figure 2B). The genetic profiles of patients in cluster I, who presented with the shortest median survival (7.5 months), were characterized by a high detection rate of *CDK4* (GenBank NG_007484) (9 of 9 [100%]), *TP53* (8 of 9 [88.9%]), *MET* (7 of 9 [77.8%]), *CDKN2A* (7 of 9 [77.8%]), *MYC* (7 of 9 [77.8%]), and *SMAD4* (GenBank NG_013013) (6 of 9 [66.7%]) (Figure 2B); moreover, *CDK4* (HR, 2.02; 95% CI, 1.03-3.96; *P* = .04), *CDK6* (HR, 2.52; 95% CI, 1.32-4.83; *P* = .005), and *MYC* (HR, 2.24; 95% CI, 1.21-4.15; *P* = .01) were found to be associated with poor outcomes in the further analysis of patients with *EGFR* variants (Figure 3).

Patients in cluster III (27 of 29 [93.1%]) and cluster IV (11 of 11 [100%]) carried *TP53* coaltered with *EGFR* variants. However, cluster III was characterized by high detection of *MET* (11 of 29 [37.9%]) and *RB1* (GenBank NG_009009) (11 of 29 [37.9%]) alterations, while cluster IV showed more *NTRK1* (9 of 11 [81.8%]) and *MYC* (6 of 11 [54.5%]) alterations without *MET* or cell cycle pathway–related genes detected. Cluster V was characterized by low *TP53* detection and moderate variant frequency of *CDK4* (11 of 26 [42.3%]) and *MYC* (6 of 26 [23.1%]) (Figure 2B). With the longest median survival (55.7 months), patients in cluster II exhibited more *ALK* fusions (8 of 19 [42.1%]) as well as a low proportion of *EGFR* variants (4 of 19 [21.1%]) and no clinically actionable variants (7 of 19 [36.8%]) (Figure 2B).

### Clinical Association of Single Alteration in CSF With Survival of Patients With an *EGFR* Variant and LM or BM

Patients in clusters I, III, IV, and V all harbored *EGFR* variants. To further explore the association of different coaltered genes with the survival of 79 patients with an activated *EGFR* variant and LM or BM, genes with a detected frequency of more than 5% in CSF were included for interactive survival analysis. Patients with *EGFR* variants coaltered with *CDK4*, *CDK6*, and *MYC* alterations had shorter median survival than patients without these alterations (*CDK4*, 11.4 months vs 19.3 months; *P* = .04; *CDK6*, 8.4 months vs 20.8 months; *P* = .005; *MYC*, 12.1 months vs 21.0 months; *P* = .01) (Figure 3A-C). Patients with *MET* alterations had median survival of 13.2 months, while those without *MET* alterations had median survival of 21.0 months (*P* = .07) (Figure 3D). In further multivariate Cox proportional hazards regression analysis, *CDK6* alteration was associated with a significantly increased risk of death (HR, 2.83; 95% CI, 1.40-5.72; *P* = .004) after adjusting for other genetic alterations and patient characteristics (Table 2).

**Table 2.** Multivariate Analysis of Survival Among Patients With an *EGFR* Variant

Characteristic	Univariate analysis		Multivariate analysis	
	HR (95% CI)	*P* value	HR (95% CI)	*P* value
Clinical features				
Age (≥55 vs <55 y)	1.23 (0.69-2.20)	.48	NA	NA
Sex (male vs female)	1.19 (0.69-2.08)	.53		NA
Smoking (yes vs no)	1.26 (0.58-2.72)	.56	NA	NA
No. of BM (0 vs ≥1)	0.58 (0.38-0.86)	.008	0.53 (0.35-0.81)	.003
ECOG PS score (4 vs 3 vs 2 vs 1)	1.54 (1.03-2.31)	.03	NA	NA
Genetic alterations				
*MET* (variant vs wild type)	1.71 (0.96-3.05)	.07	NA	NA
*MYC* (variant vs wild type)	2.24 (1.21-4.15)	.01	NA	NA
*CDK6* (variant vs wild type)	2.52 (1.32-4.83)	.005	2.83 (1.40-5.72)	.004
*CDK4* (variant vs wild type)	2.02 (1.03-3.96)	.04	1.97 (0.93-4.17)	.08

Abbreviations: BM, brain metastases; ECOG PS, Eastern Cooperative Oncology Group performance score; HR, hazard ratio; NA, not applicable.

## Discussion

To our knowledge, this study of 94 patients with advanced lung adenocarcinoma with CSF samples profiled by next-generation sequencing represents the largest reported series to date, and for the first time, we defined 5 subgroups of CNS metastases characterized by different multiple alterations that were associated with diverse outcomes. We also challenged the view that the coexistence of other variants with *EGFR* variants had the same association with the survival of patients with CNS metastases.

Extracranially, *TP53* and somatic copy number variations were associated with lung cancer.^5,6^ Concordant with previous findings, we first found that patients with CNS metastases characterized by *TP53* and somatic copy number variations in CSF had poor survival. Cell cycle pathway alterations were frequently detected and may be one of the mechanisms in BM or LM.^7^ Our study further verified that cell cycle–regulated genes were also identifiers of CNS metastases in patients with poorer survival. Likewise, *MET* copy number gain has been associated with resistance to gefitinib in LM,^8^ but we did not see a statistically significant association between *MET* alterations and inferior survival in our study. Further investigation is needed in this regard.

We identified that a subgroup (cluster I) of patients with lung adenocarcinoma and CNS metastases experienced significantly poor survival associated with the molecular characterization of their CSF samples. This finding supports the heterogeneous response to targeted therapies that is possibly due to different genetic backgrounds. The optimal strategies for this subgroup of patients with a poor prognosis are challenging, and more radical therapies—such as a tyrosine kinase inhibitor plus chemotherapy, antiangiogenic drugs, or preventive local therapy—may be taken into consideration,^9,10^ but they need further verification.

### Limitations

Our study has some limitations, including its retrospective design. In addition, treatment options based on each molecular subtype were not prospectively explored, so we could not identify the optimal treatments for patients in different clusters.

## Conclusions

Our results suggest that genetic alterations in CSF were associated with the survival of patients with advanced lung adenocarcinoma and CNS metastases. In those patients with *EGFR* variants, coalterations with *CDK4*, *CDK6*, and *MYC* were associated with poor outcomes. Thus, the use of CSF samples may facilitate risk stratifying CNS metastases into appropriate outcomes and provide a reference for further clinical study.

## Notes

Supplement. **eFigure.** Genetic Alteration Profile of Cerebrospinal Fluid Tumor DNA From All Included Patients (N = 94)
