# Supplementary material for: Association of Cerebrospinal Fluid Tumor DNA Genotyping With Survival Among Patients With Lung Adenocarcinoma and Central Nervous System Metastases
Source: JAMA Netw Open. 2020 Aug 4;3(8):e209077. doi: 10.1001/jamanetworkopen.2020.9077 (PMC7403922; doi:10.1001/jamanetworkopen.2020.9077)

Supplementary Online Content

Li Y-S, Zheng M-M, Jiang B-Y, et al. Association of cerebrospinal fluid tumor DNA genotyping with survival among patients with lung adenocarcinoma and central nervous system metastases. *JAMA Netw Open.* 2020;3(8):e209077.
doi:10.1001/jamanetworkopen.2020.9077

eFigure. Genetic Alteration Profile of Cerebrospinal Fluid Tumor DNA From All Included Patients (N = 94)

This supplementary material has been provided by the authors to give readers additional information about their work.

© 2020 Li Y-S et al. *JAMA Network Open.*

eFigure. Genetic Alteration Profile of Cerebrospinal Fluid Tumor DNA From All Included Patients (N = 94)

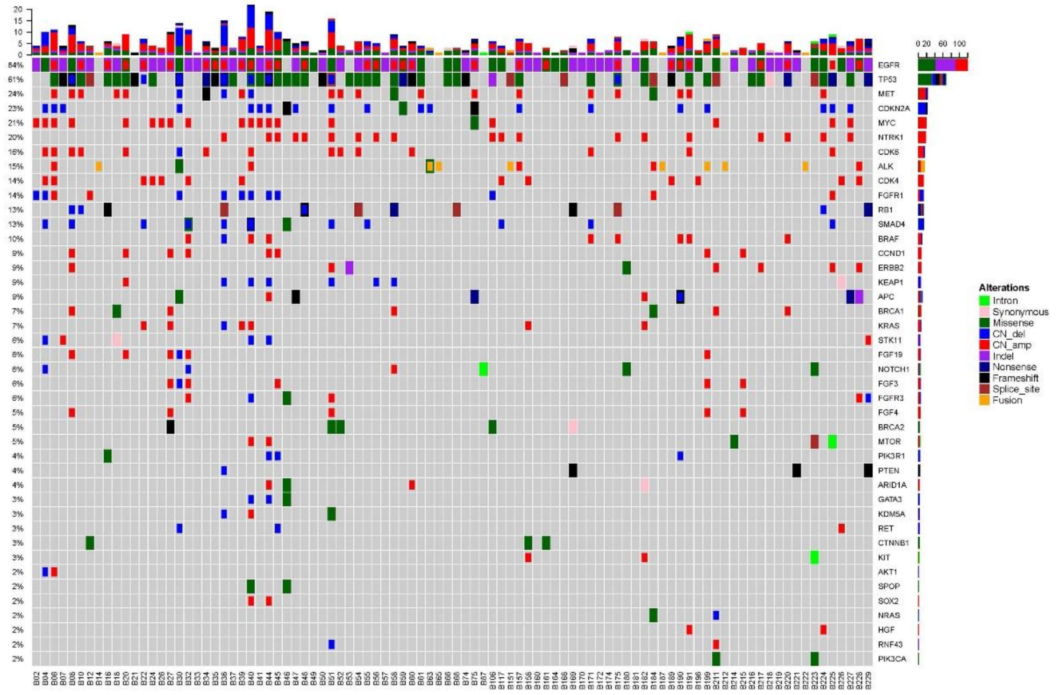

Supplement: Supplement. — eFigure. Genetic Alteration Profile of Cerebrospinal Fluid Tumor DNA From All Included Patients (N = 94) [file jamanetwopen-3-e209077-s001.pdf]
